# Development of a spontaneous model of renal interstitial fibrosis in NOD/SCID mice: Aging-induced pathogenesis

**DOI:** 10.1371/journal.pone.0315437

**Published:** 2024-12-11

**Authors:** Lihua Qiu, Zhaoxia Ma, Jinyan Li, Zhen Wu, Longmei Dai, Ruimin Long, Linlin Hu, Jianxiu Sun, Min Hu, Yanjiao Li

**Affiliations:** 1 Yunnan Key Laboratory for Basic Research on Bone and Joint Diseases, Kunming University, Kunming, China; 2 Yunnan Jici Institute for Regenerative Medicine Co., Ltd., Kunming, China; 3 Shenzhen Zhendejici Pharmaceutical Research and Development Co., Ltd., Shenzhen, China; University of Tennessee Health Science Center College of Medicine Memphis, UNITED STATES OF AMERICA

## Abstract

Renal interstitial fibrosis, a condition prevalent in aging humans and animals, is closely linked to the eventual development of renal failure. Establishing an animal model that exactly replicates the pathogenesis of renal interstitial fibrosis induced by natural aging in humans is crucial for advancing mechanistic studies and testing antifibrotic therapies. Implanted allogeneic or xenogeneic cells are cleared by the immune system when stem cell therapy is applied in nonimmunodeficient animal fibrosis models, affecting the effect of the intervention and making it difficult to demonstrate the survival, proliferation, differentiation, or secretion of the delivered autologous human-derived cells. This study effectively developed a model of spontaneous renal interstitial fibrosis linked to natural aging in 43-week-old NOD/SCID mice. Compared with those of 12- and 32-week-old mice, the kidneys of the model mice exhibited prominent fibrosis characteristics, accompanied by numerous fibrous septa and collagen deposition, increased COL1A1 expression, and decreased MMP9 expression. SA-β-gal activity and P21 gene expression levels increased, confirming renal cell senescence in the model mice. Additionally, an increase in α-SMA staining indicated an increase in epithelial–mesenchymal transition. More importantly, we observed TGF-β-SMAD3 pathway activation, mitochondrial dysfunction, decreased antioxidant capacity, oxidative stress, and an enhanced inflammatory response in the model group, consistent with renal interstitial fibrosis in elderly individuals. In this comprehensive investigation, we successfully developed a spontaneous mouse model of renal interstitial fibrosis and revealed the molecular pathways contributing to increased susceptibility to kidney injury and renal fibrosis in elderly individuals.

## Introduction

Aging is a natural progression marked by a steady diminution in cellular function and structural changes across all organ systems. Notably, the kidney is one of the organs most vulnerable to age-related damage [1]. Life expectancy is constantly increasing with the continuous progression of modern medicine, resulting in a significant increase in the number of elderly individuals. The incidence of chronic kidney disease increases with age, establishing kidney aging as the foundation for the high susceptibility and occurrence of kidney disease among elderly individuals [2,3]. Structural changes and functional decline usually occur during kidney aging, a process that typically accelerates after the age of 50–60 years [4]. These alterations manifest in various compartments within the kidney, encompassing a decrease in total nephron size and number, thickening of the glomerular basement membrane, glomerulosclerosis, and tubulointerstitial fibrosis [5]. Renal interstitial fibrosis is the most common pathway in most progressive kidney diseases, underscoring the close relationship between renal fibrosis and aging.

Renal interstitial fibrosis, also referred to as tubulointerstitial fibrosis due to its characteristic tubular destruction, represents a chronic and progressive phenomenon that affects kidney tissue as part of the aging process [6]. This abnormal fibrosis disrupts the dynamic balance of kidney structure and function, potentially leading to various end-stage renal diseases that culminate in kidney failure and threaten overall health [7]. The optimal intervention in such cases is often kidney transplantation. The pathological manifestations of renal fibrosis are renal tubular atrophy, myofibroblast accumulation, collagen deposition, and perirenal capillary exfoliation [8]. The genesis of fibrosis is primarily attributed to an imbalance between excessive synthesis and reduced decomposition of the extracellular matrix (ECM), and the accumulation of ECM serves as an early indicator of renal aging [9,10]. Myofibroblasts, generated through the process known as epithelial-to-mesenchymal transition (EMT), are responsible for producing ECM components, such as collagens and fibronectin, which originate from epithelial cells or resident interstitial fibroblasts. Research indicates that transforming growth factor β (TGF-β) is the foremost critical profibrotic cytokine capable of activating canonical Smad and other noncanonical profibrotic signaling pathways, thereby influencing the expression of numerous genes; its dysfunction has been identified as the primary cause of renal fibrosis [11]. While severe kidney fibrosis is often irreversible, early-stage fibrosis is generally considered potentially reversible. Thus, effectively managing or reversing renal fibrosis is pivotal for preventing kidney aging and mitigating the progression of numerous kidney diseases [12].

For researchers exploring fibrosis, an *in vivo* model that exactly replicates the precise pathogenesis of this condition is a critical instrument in the pursuit of elucidating underlying mechanisms and evaluating the efficacy of antifibrotic therapeutic interventions. This model facilitates mechanistic studies and forms the basis for clinical translation. A perfect animal model for renal disease research should exhibit renal anatomical, hemodynamic, and physiological characteristics akin to those of humans and be capable of indicating relevant renal, biochemical, and hemodynamic parameters [10]. Current animal models include surgical, chemical, physical, spontaneous, genetic, and *in vitro* models. Mice have emerged as more reliable tools than rats in aging research due to their genetic proximity to humans, the manipulability of their genomes, and the development of age-related phenotypes similar to those of humans throughout their lifespan [13]. Despite the time-consuming nature of following inbred mice throughout their life cycle, the longitudinal observation of these mice is ideal for aging studies. In the investigation of autologous stem cell treatment for kidney disease, beyond replicating kidney disease models with wild-type C57BL/6 mice, exploring the viability of using immunodeficient animals as alternative models for this condition has become imperative. This exploration allows for the study of human stem cell interventions within above models, with the crucial premise of circumventing immune rejection. Considering these factors, we developed a spontaneous renal interstitial fibrosis model, elicited by the natural process of aging, in Non-obese Diabetic/Severe Combined Immune-deficiency (NOD/SCID) mice and validated the model’s effectiveness using state-of-the-art measurements related to renal fibrosis. The findings of this study hold both theoretical implications and practical relevance, poised to inform and enhance the clinical strategies for addressing age-induced renal fibrosis in humans.

## Materials and methods

### Ethics statement

All animal experiments and experimental protocols were conducted in compliance with the Guidelines for Animal Care and Use of Experimental Animals of Kunming University (2021KMU011).

### Animal studies

The feeding and management methods of NOD/SCID mice were carried out as previously described [14]. In brief, in order to eliminate gender differences, the male mice were selected to be housed individually within cages under strictly controlled conditions, maintaining a 12-hour light/dark cycle with constant availability of food and water in a controlled, specific pathogen–free environment at a temperature of about 25°C, accompanied by a relative humidity of 40–60%. Euthanasia was performed when the mice reached specific age milestones:12 weeks (designated as 12 W, representing young mice, with a sample size of n = 6 and an average weight of 25.28 ± 1.44 g), 32 weeks (32 W, representing middle-aged mice, n = 6, averaging 28.53 ± 1.75 g), and 43 weeks (43 W, representing senior mice, n = 6, averaging 23.80 ± 1.12 g). Notably, the cohort of 43 W mice served as the model group, whereas the 12 W and 32 W mice collectively constituted the control groups for comparative analysis. Prior to the euthanasia procedure, the mice underwent an overnight period of fasting. Carbon dioxide (CO_2_) was used for euthanasia, ensuring a swift, painless, and stress-free demise, as CO_2_ overdose leads to swift unconsciousness followed by death. Kidney tissues were meticulously and completely removed, preserved in different ways, and prepared for subsequent analysis.

### Kidney histology evaluation

After fixation in a 4% paraformaldehyde solution, renal tissues from different groups underwent a dehydration protocol involving graded alcohol solutions. Subsequently, the tissues were embedded in paraffin and sliced into 3-μm sections. The paraffin sections were deparaffinized with xylene and then hydrated with gradient ethanol to facilitate hematoxylin and eosin (H&E) staining. The sections were subjected to Masson’s trichrome staining to assess collagen deposition in the renal interstitium for interstitial fibrosis. Images of the stained sections were acquired using an automated slide scanning system (VS200, Olympus, Tokyo, Japan). Semiquantitative, blinded scoring for tubular vacuolation was conducted according to the Banff 2018 classification [15], the evaluation criteria are as follows: 0—No tubular atrophy; 1—Tubular atrophy involving up to 25% of the area of cortical tubules; 2—Tubular atrophy involving 26 to 50% of the area of cortical tubules; 3—Tubular atrophy involving in >50% of the area of cortical tubules. The percentage of glomerular atrophy was observed in at least 10 fields and calculated by three independent researchers. The proportion of collagen deposition, represented by the blue-stained area, was calculated in relation to the total field area on each slide. This analysis was performed in a double-blinded fashion by utilizing the ImageJ software (National Institutes of Health, Bethesda, MD, USA) based on three randomly chosen regions from each slide.

### Immunohistochemistry

Dewaxing in xylene and dehydration in graded alcohol solutions were performed on 5-μm paraffin sections from each group. Subsequently, the sections were exposed to a solution of 3% H_2_O_2_ in PBS for 10 minutes at room temperature (22–25°C) to eliminate endogenous peroxidase. Then, the sections were incubated in boiling sodium citrate buffer and blocked with 2% bovine serum albumin. This was immediately succeeded by an overnight incubation at 4°C within a humidified chamber, utilizing a primary polyclonal rabbit anti-alpha-smooth muscle actin (anti-α-SMA; 1:100; ab5694; Abcam, Cambridge, MA, USA). Thereafter, they were exposed to a biotinylated secondary antibody for about 1 hour. For the visualization of α-SMA expression, 3,3’-Diaminobenzidine tetrahydrochloride (DAB) was used, resulting in the development of a distinct brown staining. Additionally, the slices were counterstained with hematoxylin to clearly delineate the nuclei. The brown-stained positive areas were captured through the automated slide scanning system. Immunohistochemical quantification of α-SMA in five fields of the renal cortex in each section was performed in a blinded manner using ImageJ software.

### Renal hydroxyproline assessment

After the mice were sacrificed, renal hydroxyproline content was evaluated using a hydroxyproline detection kit (Nanjing Jiancheng Bioengineering Institute, Nanjing, China) based on the manufacturer’s instructions. The corresponding segments of snap-frozen kidneys (200 mg) were hydrolyzed at 120°C for 2 h. The resulting supernatants were transferred to a 96-well plate, and the wells were allowed to dry. The optical density (OD) measurements were performed in triplicate at a wavelength of 550 nm, utilizing a Multiskan Spectrum (Tecan Austria GmbH, Grodig, Austria). The results were determined using a standard curve and are presented in micrograms per milligram of wet kidney tissue.

### Detection of senescence-associated β-galactosidase activity

Senescence-associated β-galactosidase (SA-β-gal) staining was conducted using a senescence detection kit (Beyotime, Shanghai, China) following the manufacturer’s instructions. Briefly, the kidneys were rapidly frozen, embedded in optimal cutting temperature compound (Sakura Finetek USA, Torrance, CA, USA), and cut into 10-μm sections using a cryostat microtome (Thermo Fisher Scientific, Waltham, MA, USA). Afterward, the obtained cryosections were washed with PBS three times and incubated with β-Gal staining solution overnight at 37°C. The occurrence of SA-β-gal-positive cells was evaluated utilizing the automated slide scanning system. To quantify the expression of β-Gal in the different groups, we calculated the density of blue in five fields in each section using ImageJ software.

### Transmission electron microscopy

Transmission electron microscopy (TEM) analysis was conducted as previously described [14]. In brief, kidney tissues were fixed in 2.5% glutaraldehyde buffered in 0.1 mol/L phosphate buffer at pH 7.2. The specimens were then immersed in 1% osmium tetroxide for 2 hours, serially dehydrated in ethanol, and embedded in SPI-Pon 812 resin, followed by polymerization at 60°C for 48 hours. For further examination, ultrathin slices of 60 nm thickness were precisely cut utilizing a Leica EM UC7 ultracutmicrotome (Wetzlar, Germany) and then subjected to staining with 2% uranyl acetate, followed by lead citrate. These prepared slices were examined under the high-resolution lens of a JEM-1400 Plus transmission electron microscope, functioning at an energy level of 80 kV (Japan Electron Optics Laboratory Co., Ltd., Tokyo, Japan).

### RNA extraction and real-time PCR

Total RNA was isolated from kidney tissues with TRIzol (Invitrogen, Carlsbad, CA, USA). cDNA was subsequently synthesized using a cDNA synthesis kit (Takara, Dalian, China). The expression levels of crucial genes were assessed via qPCR using the QuantStudio Q5 System (Applied Biosystems, Waltham, MA, USA). The primers for qPCR were devised using Primer Premier 5.0 [16], are detailed in S1 Table. The qPCR assays were conducted using Dib^®^ SYBR qPCR SuperMix Plus (Hong Kong Aibisheng Biotechnology Co., Ltd., China). Relative expression levels were quantitatively assessed adopting the 2^–ΔΔCt^ methodology, with ACTB mRNA serving as an internal control. Detailed qPCR results for all genes are provided in S2 Table.

### Statistical analysis

Quantitative data are presented as the mean ± SEM for each group. Group comparisons were conducted using one-way ANOVA, with significance determined at a threshold of P < 0.05. Statistical analyses and the generation of graphical illustrations were conducted utilizing GraphPad Prism 5 (GraphPad Software Inc., La Jolla, CA, USA).

## Results

### Effects of aging on renal interstitial fibrosis in mice

**Fig 1:**
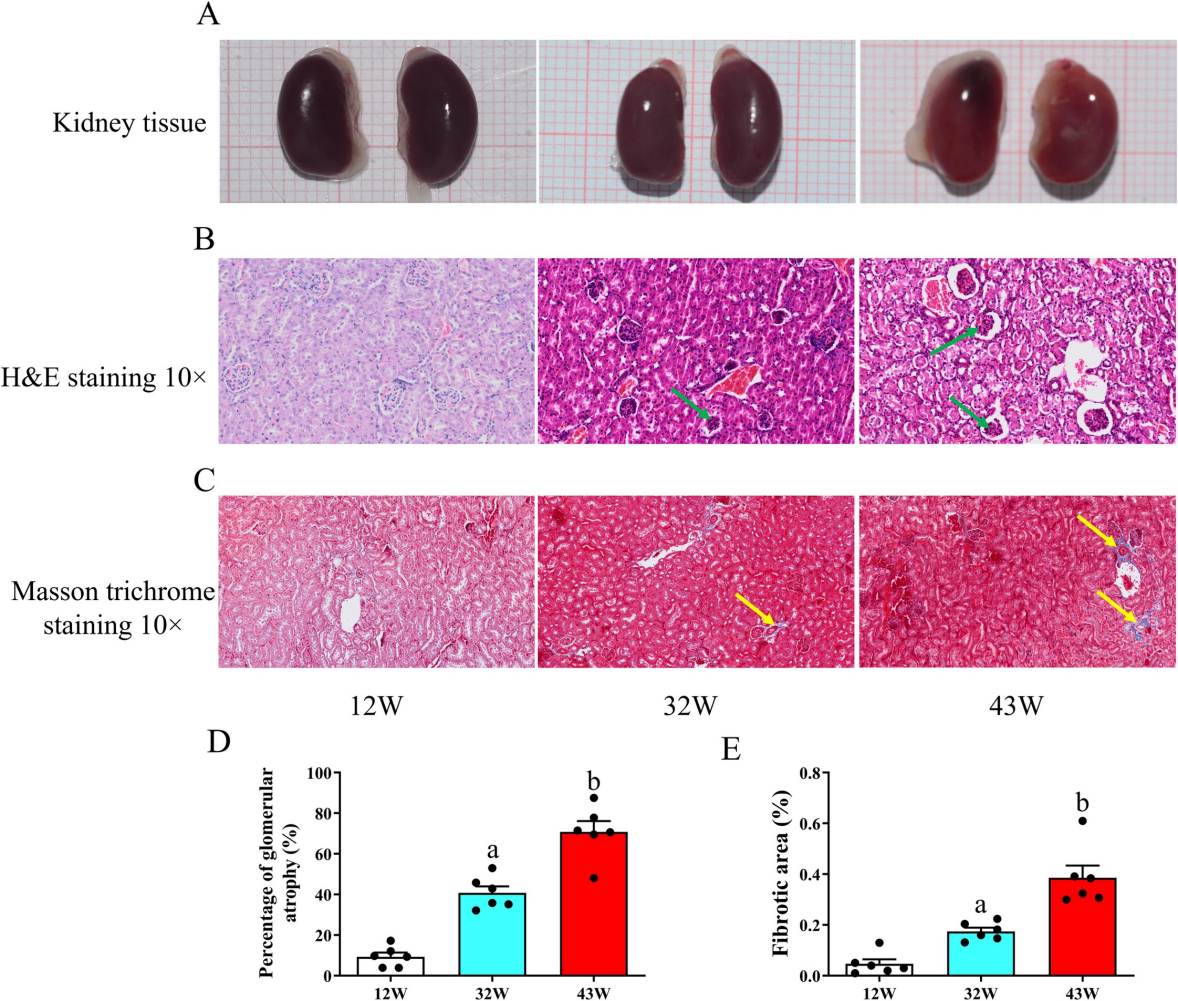
Alterations in renal tubular morphology and renal interstitial collagen deposition were observed in aged mice. (A) Visual changes of the kidneys of the experimental cohort. (B) Morphological assessment of kidney slices subjected to H&E staining (10×). (C) Masson’s trichrome staining was employed at 10× magnification to detect collagen fibers. (D) Quantitative analysis of the percentage of renal tubular atrophy. (E) Quantitative analysis of the relative density of positive areas in renal tubules, indicating changes in collagen deposition. Data are displayed with the mean ± SEM, with distinct letters (a–b) indicating statistically significant variations (P < 0.05) compared with 12 W. Tubular atrophy and collagen deposition are highlighted by green and yellow arrows, respectively.

On visual observation, the color of kidney tissue of mice at 12 W was dark red, but the color gradually faded at 32 W and turned light red at 43 W, indicating varying degrees of tissue damage (Fig 1A). Renal tubular morphological changes in mice of different ages were subsequently assessed employing H&E and Masson’s trichrome staining. H&E staining unveiled normal renal tubule structure with minimal exfoliation of epithelial cells in the 12 W group, with a vacuolization score of 0 (Fig 1B). In contrast to the 12 W group, the other two groups, especially in the 43 W group, exhibited severe pathological changes, including renal tubular atrophy, vacuolar lesions, and exfoliation of epithelial cells, where the vacuolization scores were 1 and 3, respectively. The tubular atrophy rate, which also increased with age, was consistent with these findings (Fig 1D). Masson’s trichrome staining indicated that the kidney tubules in the 12 W group had fewer collagen fibers than those in the 32 W and 43 M groups; the 43 M group, in particular, demonstrated a substantially larger number of collagen fibers than the 12 W group (Figs 1C and 1E and S1).

### Effect of renal aging on EMT and collagen in mice

**Fig 2:**
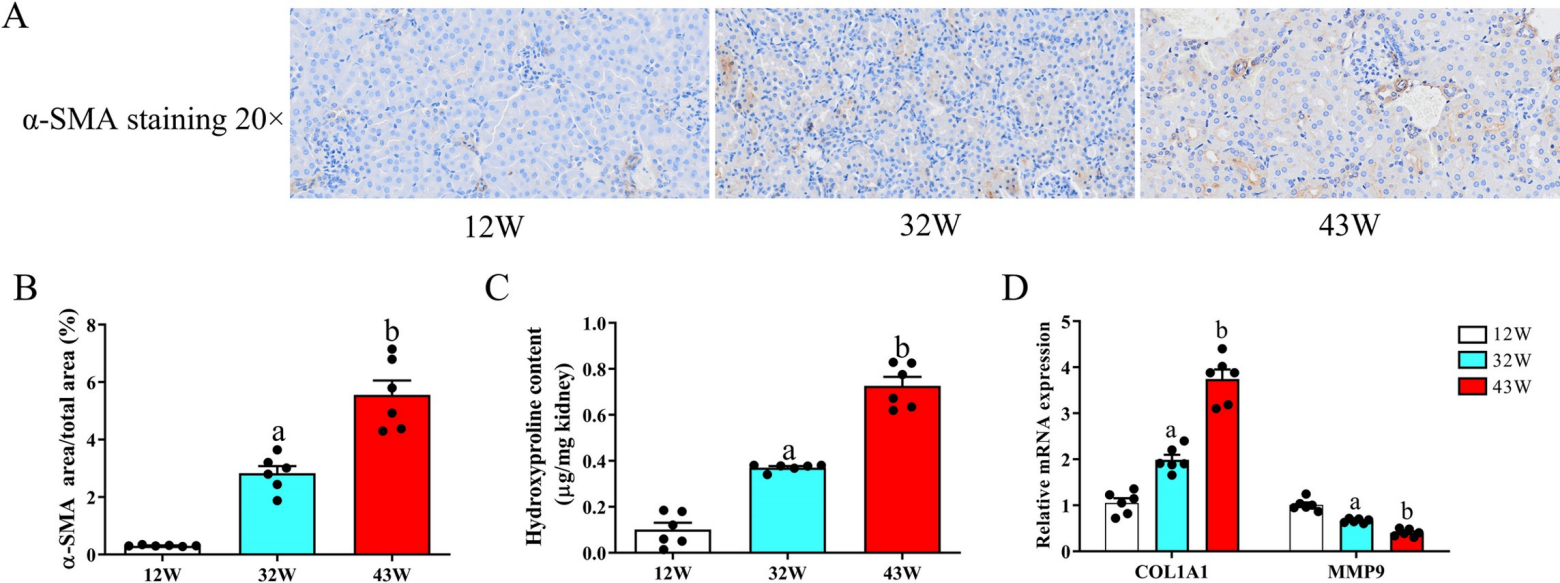
Aging enhances α-SMA expression and collagen accumulation in the kidney. (A) The expression of α-SMA in renal tubules at 20× magnification. (B) Quantitative analysis of the relative expression of α-SMA in renal tubules. (C) Quantitative analysis of Hydroxyproline content within kidney tissue. (D) The mRNA abundance of the COL1A1 and MMP9 genes within kidney tissue. Data are displayed with the mean ± SEM, with distinct letters (a–b) indicating statistically significant variations (P < 0.05) compared with the findings in the 12 W group.

To further investigate interstitial fibrosis induced by natural aging, we performed immunohistochemical analyses targeting myofibroblast marker α-SMA, which is also commonly used for evaluating EMT. Immunohistochemical staining revealed minimal α-SMA staining in the kidneys of 12 W mice, whereas α-SMA staining in the 32 W and 43 W groups exhibited a marked increase compared with that in the 12 W group (P < 0.05), with the most staining detected in the kidneys of mice in the 43 W group (Figs 2A and 2B and S2). Concurrently, the hydroxyproline content was evaluated within kidney tissues and noted a substantial increase in the 32 W and 43 W groups in comparison to the 12 W group (P < 0.05), particularly in the 43 W group (Fig 2C). Quantitative analysis of COL1A1 expression yielded results analogous to those of hydroxyproline (P < 0.05) (Fig 2D). In addition, aging resulted in a marked decrease in MMP9 mRNA expression (P < 0.05).

### Effect of aging on cellular senescence in the mouse kidney

**Fig 3:**
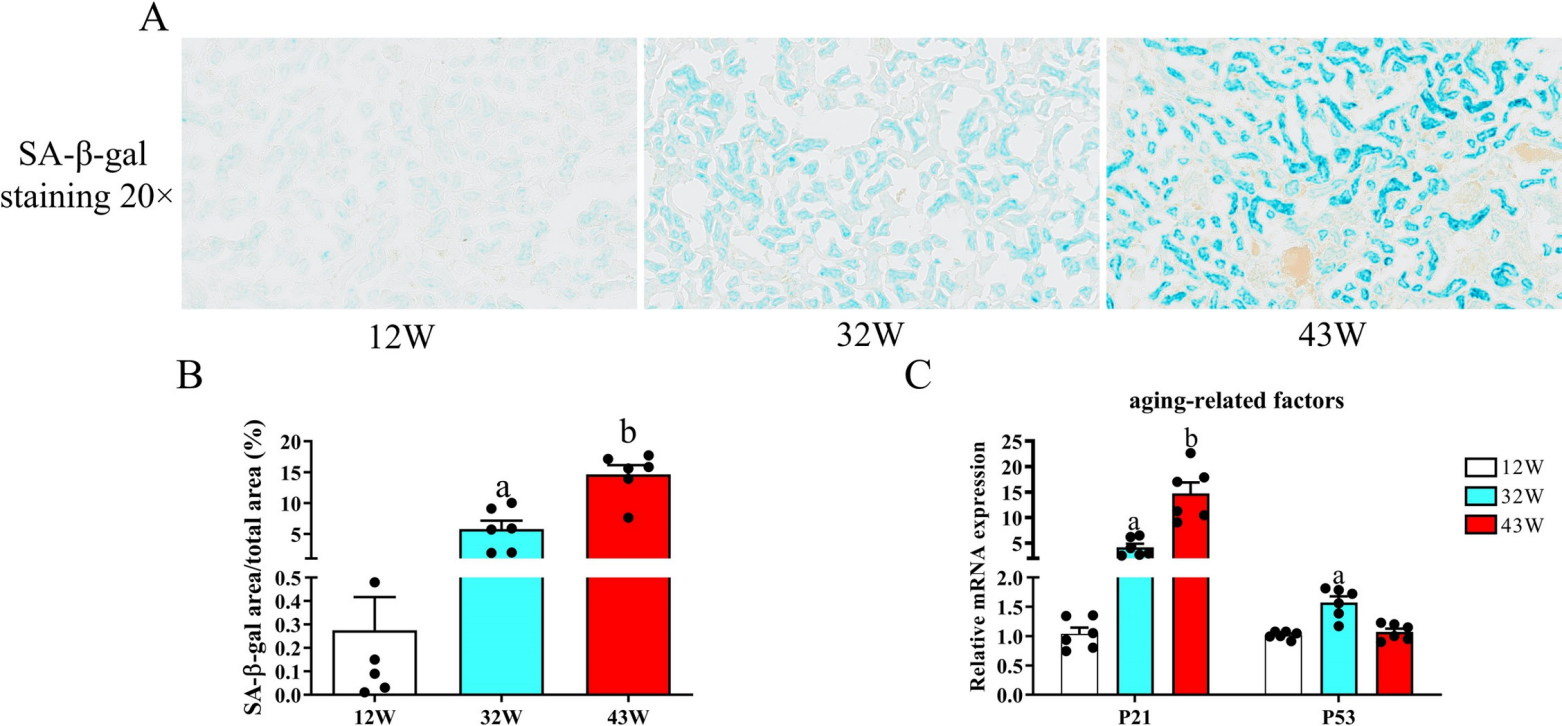
Effect of aging on cellular senescence in the mouse kidney. (A) SA-β-gal staining (displayed in turquoise blue) of kidney slices from mice of varying ages is presented at 20× magnification. (B) The mean density of SA-β-Gal within the renal cortex. (C) Quantitative real-time PCR analysis of the mRNA levels of the aging-associated genes P21 and P53 in the kidney. Data are displayed with the mean ± SEM, with distinct letters (a–b) indicating statistically significant variations (P < 0.05) compared with the findings in the 12 W group.

SA-β-gal is often used as a marker of cell senescence; thus, we detected its activity in the kidneys of mice across various age groups. The number of SA-β-gal-positive mice was significantly greater in the 32 W group than in the 12 W group (P < 0.05), with this augmentation persisting into the 43 W group as well (P < 0.05) (Figs 3A and 3B and S3). Subsequently, we delved deeper into the mechanisms underlying aging in the kidney by investigating the expression patterns of two crucial aging-associated factors P21 and P53 in the kidney via qPCR. The results showed a significant increase in P21 expression in 32 W and 43 W mice, in stark contrast to the levels observed in 12 W mice (P < 0.05), as depicted in Fig 3C. Compared with that in 12 W mice, renal P53 mRNA expression markedly increased in 32 W mice (P < 0.05) but did not significantly change in 43 W mice (P > 0.05).

### Impaired mitochondrial function and antioxidant deficiency in the kidneys

**Fig 4:**
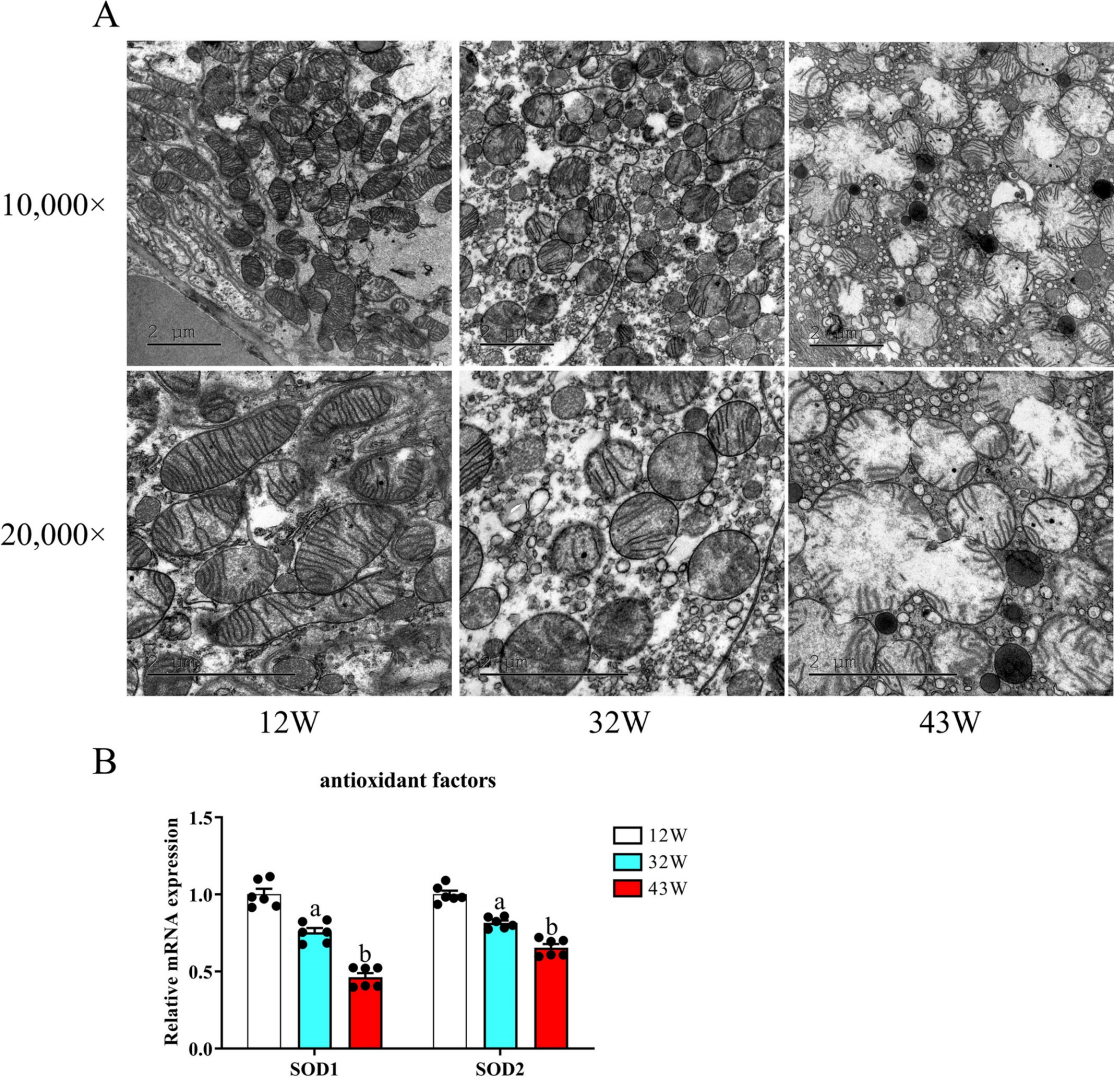
Aging impairs renal mitochondrial function and antioxidant capacity. (A) Representative transmission electron microscopy images of kidney tissue at 10,000× and 20,000× magnification. (B) Quantitative real-time PCR analysis of the antioxidant-associated genes SOD1 and SOD2. Data are displayed with the mean ± SEM, with distinct letters (a–b) indicating statistically significant variations (P < 0.05) compared with the observations in the 12 W group (P < 0.05).

Renal aging involves various mechanisms, with mitochondrial dysfunction being a key contributor [17]. We used transmission electron microscopy (TEM) to assess morphological changes in mitochondria. Electron microscopic analysis revealed normally shaped and elongated mitochondria with intact cristae in 12 W mice (Fig 4A). In contrast, the mitochondrial structure was compromised in 32 W mice, exhibiting heterogeneous sizes, distorted shapes, and partial erosion of mitochondrial cristae. These degenerative changes became even more pronounced in 43 W mice, characterized by the emergence of cytoplasmic vacuoles, extensive disruption of mitochondrial morphology, and the complete loss of mitochondrial cristae. We then delved into the transcriptional consequences of aging on the antioxidant factors SOD1 and SOD2. SOD1 and SOD2 expression was significantly lower in 32 W and 43 W mice than in 12 W mice, especially in 43 W mice (P < 0.05) (Fig 4B).

### Changes in mRNA abundance of renal fibrosis-related genes in aged mice

**Fig 5:**
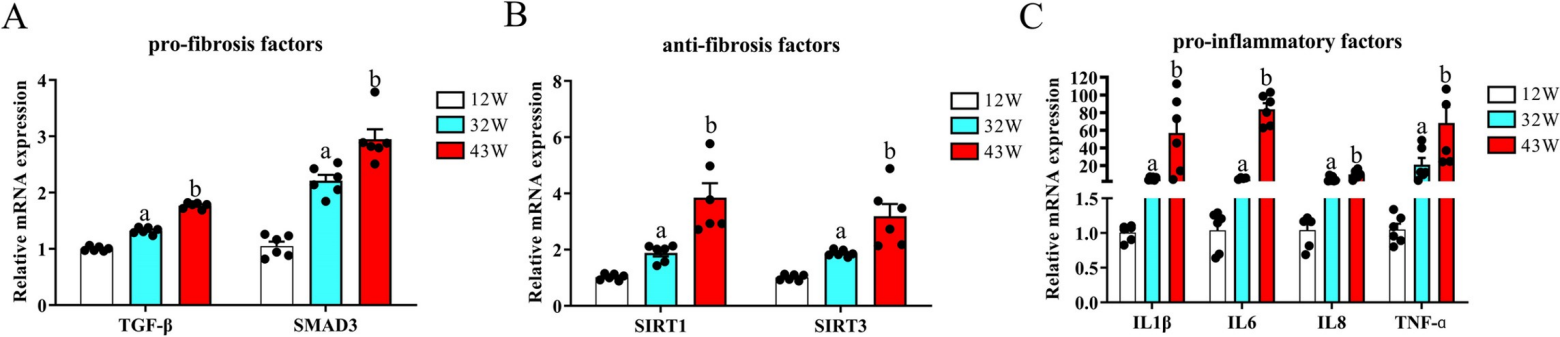
Aging regulates mRNA levels of renal fibrosis-related genes in mice across varying age cohort. (A) Changes in mRNA levels of genes associated with profibrosis in 12 W, 32 W, and 43 W mice. (B) Changes in mRNA levels of genes associated with antifibrosis activity in 12 W, 32 W, and 43 W mice. (C) Changes in mRNA levels of genes associated with inflammation in 12 W, 32 W, and 43 W mice. Data are displayed with the mean ± SEM, with distinct letters (a–b) indicating statistically significant variations (P < 0.05) compared with those observed in the 12 W group (P < 0.05).

To further investigate renal fibrosis-related factors, we analyzed the mRNA abundance of coding genes associated with this process. Our results revealed that among the profibrosis-related genes, TGF-β and SMAD3 were expressed at lower levels in 12 W mice. However, the expression of these genes significantly elevated in 32 W and 43 W mice (P < 0.05), indicating an age-related increase (Fig 5A). Notably, SIRT1 and SIRT3, considered antifibrotic factors, also exhibited an age-related enhancement in their expression profiles (P < 0.05) (Fig 5B). The expression trends of the proinflammatory factor-encoding genes IL1β, IL6, IL8, and TNF-α were consistent; that is, there was a weak presence in the kidneys of 12 W mice, while in 32 W and notably 43 W mice, their expression significantly increased in the kidney compared with that in 12 W mice (P < 0.05) (Fig 5C).

## Discussion

CKD has emerged as a global health concern, affecting roughly 30–40% of patients who progress to end-stage renal disease within 10–15 years of diagnosis. Renal fibrosis is the principal pathological mechanism driving CKD progression, leading to impaired renal function [18]. Renal fibrosis is a prevalent condition that affects nearly 10% of the global population and contributes to elevated mortality rates and a strain on national healthcare systems [19]. The absence of translational models for human renal fibrosis poses a significant challenge to the development of effective antifibrotic therapies [20]. Due to the lack of cellular heterogeneity in *in vitro* models, animal models are indispensable for exploring the pathobiology of renal fibrosis and assessing novel treatment modalities. Aging is a pivotal factor intricately linked to the initiation and progression of renal fibrosis. Aging-induced renal interstitial fibrosis in humans has progressed over the past several decades and involves the gradual cross-linking of collagen, which confers resistance to degradation and distorts the architecture of renal tubules. Consequently, establishing a suitable spontaneous animal model for renal fibrosis induced by aging has become imperative. Such a model holds profound significance for addressing early renal fibrosis in the aging process, particularly in the context of stem cell interventions aiming to mitigate the incidence of age-related kidney diseases. Notably, spontaneous renal fibrosis models have been successfully developed in rat models, such as Buffalo/Mna and Munich Wistar Frömter rats [10]. However, the development of a spontaneous renal fibrosis model has not been reported in NOD/SCID mice.

In this study, we established a spontaneous renal fibrosis model by examining alterations in renal morphology and function in NOD/SCID mice across different age groups while concurrently assessing the effect of aging on renal fibrosis. The histological and pathophysiological findings of this study aligned with those observed in aging C57BL/6 mice and KM mice [21,22], which exhibit morphological characteristics similar to those of mouse models of renal interstitial fibrosis induced by hypoxia and unilateral ureteral obstruction (UUO), characterized by tubular atrophy and collagen deposition [23,24]. The pathological progression of renal fibrosis involves four distinct stages: (1) an initiation stage involving cell activation and injury; (2) an activation stage marked by the initiation of fibrotic signaling pathways; (3) a fibrosis stage characterized by ECM accumulation; and (4) a progressive stage of fibrosis [25]. Throughout renal fibrosis development, injured kidney cells of almost all origins undergo phenotypic loss and transdifferentiate into myofibroblasts through EMT, resulting in excessive production and deposition of ECM [11]. Myofibroblasts express α-SMA and contribute to collagen deposition; therefore, α-SMA serves as a key marker for myofibroblasts in the kidney, and changes in its expression can reflect the progression of EMT [26]. Dysregulation of TGF-β-driven signaling is the main cause of EMT and renal fibrosis, and in particular, blocking the TGF-β/Smad3 pathway substantially attenuates fibrosis in animal models of kidney disease [27]. In the present study, renal interstitial fibrosis induced by natural aging was accompanied by significant EMT progression and collagen accumulation. This effect was evident through a substantial increase in the expression of α-SMA and COL1A1, consistent with UUO-induced renal fibrosis [24]. Additionally, aging increased the mRNA levels of TGF-β and SMAD3, concurrently inhibiting MMP9, an endopeptidase responsible for ECM degradation. Previous research has shown that TGF-β can inhibit MMPs, further promoting ECM production, consistent with the outcomes of this study. The observed increase in hydroxyproline content in the renal tissues of aged mice in this study further substantiates collagen accumulation.

Various factors contribute to aging, including cellular senescence, inflammation, telomere dysfunction, and metabolic changes, culminating in age-related renal fibrosis [5]. Senescent cell accumulation actively participates in renal fibrosis development by upregulating proinflammatory mediators and profibrotic factors, ultimately impeding cell rejuvenation [28]. Simultaneously, inflammatory responses play a crucial role in fibrosis development. While normal wound healing-associated inflammation resolves without additional tissue damage, chronic conditions sustain unresolved inflammation, leading to a fibrotic response and decreased tissue function. Persistent inflammation, if unresolved, triggers the production of profibrotic cytokines and growth factors by kidney cells involved in the inflammatory response and infiltrating immune cells [5]. At the subcellular level, intricate interactions among these profibrotic cytokines and growth factors culminate in progressive interstitial fibrosis. The tumor suppressor gene P53 coordinates oxidative stress and DNA damage responses, simultaneously promoting P21 activation and resulting in cell cycle arrest and senescence [29]. The sirtuin (SIRT) family plays a key role in regulating various biological and cellular processes in the aging process [30], including SIRT1, which influences the oxidative response and cell cycle regulation by modifying the activity of target proteins in the nucleus, and SIRT3, which is closely associated with mitochondrial oxidative stress [31]. Previous studies have shown that increased renal mRNA expression of P53 and P21 in mice induced by aristolochic acid is accompanied by augmented SA-β-gal activity [1]. In our study, similar results were obtained in aged mice, except for the anticipated increase in P53 gene expression in 43 W mice. Cell aging is accompanied by an inflammatory response, and the continued activation of the inflammation-driven NF-κB pathway significantly inhibits P53 function [32]. In addition, due to the presence of EMT during the process of renal fibrosis, there are a large number of activated myofibroblasts [33], which may also be the reason for the decrease in P53 gene expression in 43 W mice. Whether this is the case requires further experimental verification. In contrast to previous research on old mice reporting a significant decrease in SIRT1 expression [22], our study revealed an increase in SIRT1 and SIRT3 expression with age. We postulate that there is a potential link between enhanced lipid metabolism during aging [34] and the involvement of SIRT1 in lipid metabolism [13]; however, this hypothesis requires further experimental verification. Regarding proinflammatory cytokines, studies on older SD rats confirmed that IL-19 and IL-24 expression levels were elevated in the kidney [35] and that TNF-α also contributed significantly to fibrosis pathogenesis [36]. Similarly, our study highlighted a robust inflammatory response in a naturally aging mouse model of renal fibrosis, as evidenced by the increased expression of multiple proinflammatory factors with age.

Cell senescence directly leads to mitochondrial dysfunction, which drives and maintains cell senescence [1]. Renal fibrosis is characterized by numerous pathological features rooted in reduced ATP production, a consequence of mitochondrial dysfunction characterized by increased free radical generation and oxidative stress [33]. The increase in reactive oxygen species (ROS) formation is not merely an outcome of renal fibrosis but a positive influence on profibrotic signaling. Oxidative stress plays a pivotal role in the etiology of renal fibrosis, stemming from an imbalance between heightened ROS production and reduced antioxidant defenses. Perturbation of the cellular antioxidant system further affects downstream signal transduction, contributing to renal senescence, apoptosis, fibrosis, and renal cell regeneration. Research on aged SD rats corroborates the decline in renal total antioxidant capacity and MnSOD protein expression in the renal cortex and medulla compared with their younger counterparts [37]. Coincidentally, in this study, the mitochondrial structure of the kidney was seriously damaged in aging NOD/SCID mice, and mRNA levels of SOD1 and SOD2 was also markedly inhibited. This indicates that the antioxidant capacity of the kidneys of these mice decreased, which is consistent with the observed decrease in total SOD activity and SOD1 and SOD2 protein expression in aging C57BL/6 mice [22]. Consequently, considering the comprehensive findings of this study, we successfully established a spontaneous renal interstitial fibrosis model in 43 W NOD/SCID mice. This model is beneficial for assessing the survival, proliferation, and differentiation of human cells *in vivo* after autologous cell transplant in the study of renal fibrosis.

## Conclusions

This study successfully developed a model illustrating spontaneous renal interstitial fibrosis resulting from natural aging in NOD/SCID mice. The model, which was meticulously assessed through morphological observations and measurements of relevant fibrosis indicators, remarkably mirrored established models of renal interstitial fibrosis generated through alternative methodologies. The findings were consistent with the characteristics observed in human renal interstitial fibrosis. Furthermore, the established mouse model of renal interstitial fibrosis revealed the molecular pathways underlying increased susceptibility to renal injury and fibrosis in elderly individuals. These insights hold promise for the formulation of targeted treatment strategies.

## Supporting information

S1 Table Primers used for qPCR. (PDF)

S2 Table The qPCR results of all related genes in mice. (PDF)

S1 Fig Results of masson’s trichrome staining in all mice. (PDF)

S2 Fig Results of α-SMA staining in all mice. (PDF)

S3 Fig Results of SA-β-gal staining in all mice. (PDF)
