# Supplementary material for: Development of a spontaneous model of renal interstitial fibrosis in NOD/SCID mice: Aging-induced pathogenesis
Source: PLoS One. 2024 Dec 11;19(12):e0315437. doi: 10.1371/journal.pone.0315437 (PMC11633998; doi:10.1371/journal.pone.0315437)
Supplement: S1 Table — (PDF) [file pone.0315437.s001.pdf]

S1 Table Primers used for qPCR

Genes	Primer sequences (5'-3')
COL1A1	F: CAAGGGTGAGACAGGCGAACAAG<br>R: GAGAACCAGGAGAACCAGGAGGAC
MMP9	F: CCCATGTCACTTTCCCTTCACCTTC<br>R: CGTTGCCGTGCTCCGTGTAG
P21	F: CCTTGTCGCTGTCTTGCACTCTG<br>R: GCTGGTCTGCCTCCGTTTTCG
P53	F: CACAGCACATGACGGAGGTC<br>R: TCCTTCCACCCGGATAAGATG
SIRT1	F: GACCTCCCAGACCCTCAAGCC<br>R: GTGACACAGAGACGGCTGGAAC
SIRT3	F: GGCTCTATACACAGAACATCGAC<br>R: TAGCTGTTACAAAGGTCCCGT
IL1β	F: TGGCAACTGTTCCTGAACTCAACTG<br>R: TCATCTTTTGGGGTCCGTCAACTTC
IL6	F: TAGTCCTTCCTACCCCAATTTCC<br>R: TTGGTCCTTAGCCACTCCTTC
IL8	F: ACCTGCTCTGTCACCGATGTCTAC<br>R: CAGGCAAGGTCAGGGCAAAGAAC
TNF-α	F: CAGGCGGTGCCTATGTCTC<br>R: CGATCACCCCGAAGTTCAGTAG
TGF-β	F: ACAATGGCGGTGCGGTCAAG<br>R: CAGACTTCATGCGGCTTCTCACAG
SMAD3	F: CACGCCTCACAGCCATCCATG<br>R: TCCCAATGTGTCGCCTTGTAAGTTC
SOD1	F: AGAGCATTCCATCATTGGCCGTAC<br>R: CGCAATCCCAATCACTCCACAGG
SOD2	F: CAATCTCAACGCCACCGAGGAG<br>R: AGGGCTCAGGTTTGTCCAGAAAATG
ACTB	F: GTGACGTTGACATCCGTAAAGA<br>R: GCCGGACTCATCGTACTCC
