# Supplementary material for: Development of a spontaneous model of renal interstitial fibrosis in NOD/SCID mice: Aging-induced pathogenesis
Source: PLoS One. 2024 Dec 11;19(12):e0315437. doi: 10.1371/journal.pone.0315437 (PMC11633998; doi:10.1371/journal.pone.0315437)
Supplement: S2 Table — (PDF) [file pone.0315437.s002.pdf]

S2 Table The qPCR results of all related genes in mice

Genes	Mouse group																	
	12W						32W						43W					
COL1A1	0.71	0.81	1.22	1.35	1.14	1.05	2.40	1.66	1.91	1.88	1.9	2.2	3.88	3.19	4.02	3.1	4.4	3.88
MMP9	1.24	0.94	1.03	0.86	0.95	1.01	0.71	0.63	0.64	0.68	0.61	0.72	0.34	0.39	0.48	0.42	0.51	0.32
P21	1.34	1.35	0.74	0.80	1.05	0.93	4.02	2.56	2.98	6.21	6.51	2.75	10.49	9.09	11.26	17.88	22.67	17.03
P53	1.05	0.91	0.99	1.00	1.08	1.08	1.82	1.56	1.79	1.72	1.39	1.17	1.00	1.23	0.90	0.95	1.15	1.20
SOD1	0.92	0.92	0.97	0.98	1.12	1.10	0.68	0.68	0.75	0.76	0.83	0.82	0.52	0.52	0.52	0.41	0.40	0.40
SOD2	0.98	0.98	0.98	1.04	1.09	0.94	0.85	0.82	0.80	0.78	0.77	0.86	0.61	0.60	0.61	0.69	0.70	0.72
TGF-β	0.97	1.06	1.04	0.97	0.96	1.00	1.35	1.29	1.38	1.38	1.31	1.23	1.78	1.79	1.72	1.69	1.81	1.82
SMAD3	0.82	0.94	0.88	1.23	1.26	1.16	2.14	1.84	2.05	2.42	2.28	2.53	3.79	2.88	2.82	2.79	2.89	2.51
SIRT1	1.00	0.88	1.15	1.13	0.93	1.06	2.12	2.02	2.02	2.13	1.57	1.43	2.71	3.78	2.93	2.93	5.77	4.97
SIRT3	1.10	0.89	1.09	0.94	1.12	0.92	2.03	1.98	1.83	1.81	1.72	1.85	2.14	2.73	2.17	4.89	3.47	3.73
IL1β	0.83	0.90	1.05	1.10	1.07	1.08	3.46	3.67	3.81	7.12	6.61	7.08	13.99	45.57	14.17	73.14	92.27	112.74
IL6	1.26	1.14	0.70	0.64	1.29	1.21	2.80	4.20	3.78	6.29	5.39	6.02	62.62	80.37	65.05	92.35	103.20	98.64
IL8	1.17	1.16	1.22	0.69	1.20	0.81	2.63	2.57	3.03	3.27	7.66	9.07	7.11	6.94	13.53	16.34	10.09	10.46
TNF-α	1.22	0.80	1.34	1.09	0.96	0.89	12.50	13.13	19.61	12.38	28.63	29.89	24.64	24.20	36.98	106.66	89.26	127.33
