# Supplementary figures and images for: Development of a spontaneous model of renal interstitial fibrosis in NOD/SCID mice: Aging-induced pathogenesis
Source: PLoS One. 2024 Dec 11;19(12):e0315437. doi: 10.1371/journal.pone.0315437 (PMC11633998; doi:10.1371/journal.pone.0315437)

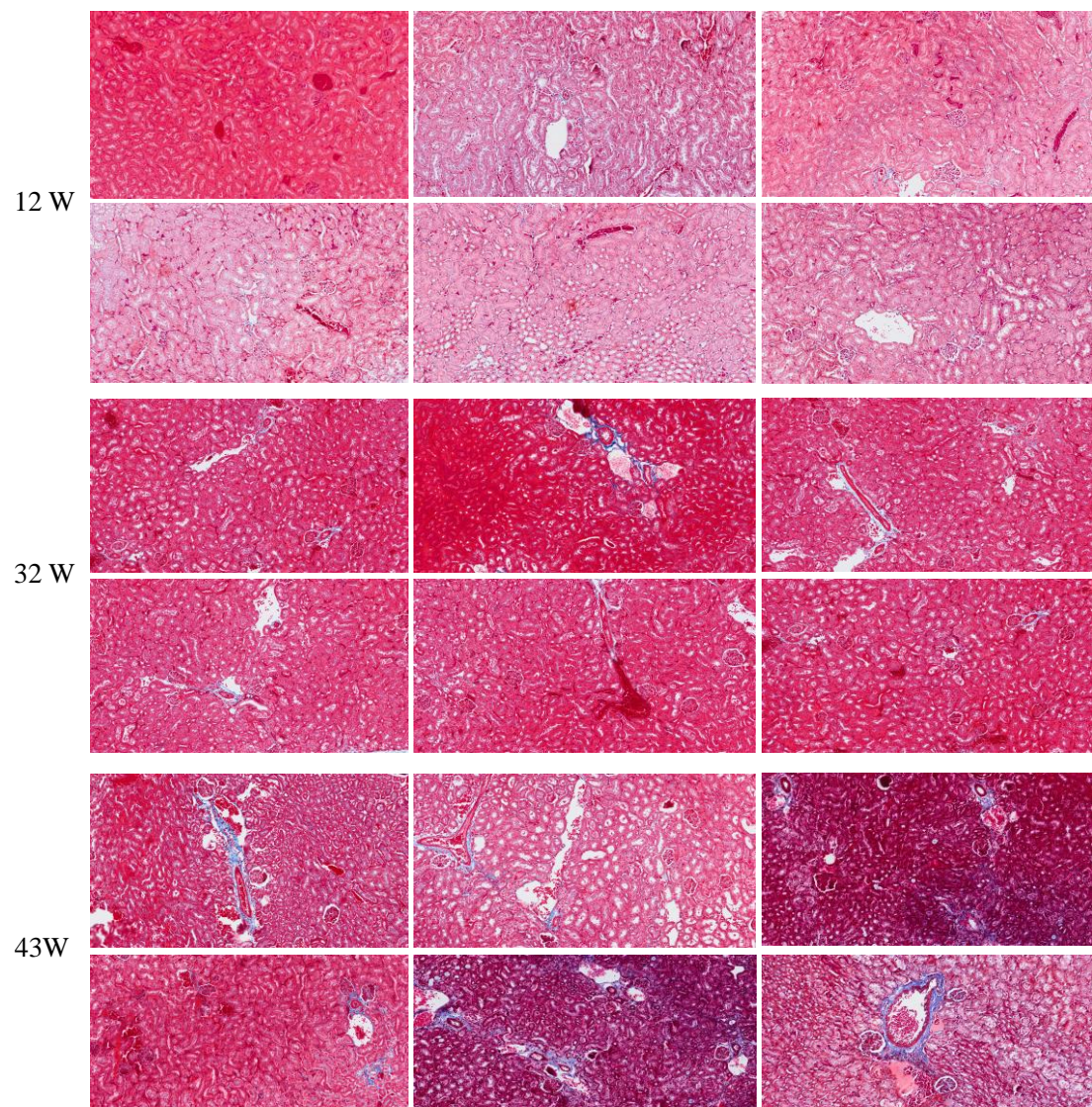

**S1 Fig. Results of masson's trichrome staining in all mice.**

Supplement: S1 Fig — (PDF) [file pone.0315437.s003.pdf]

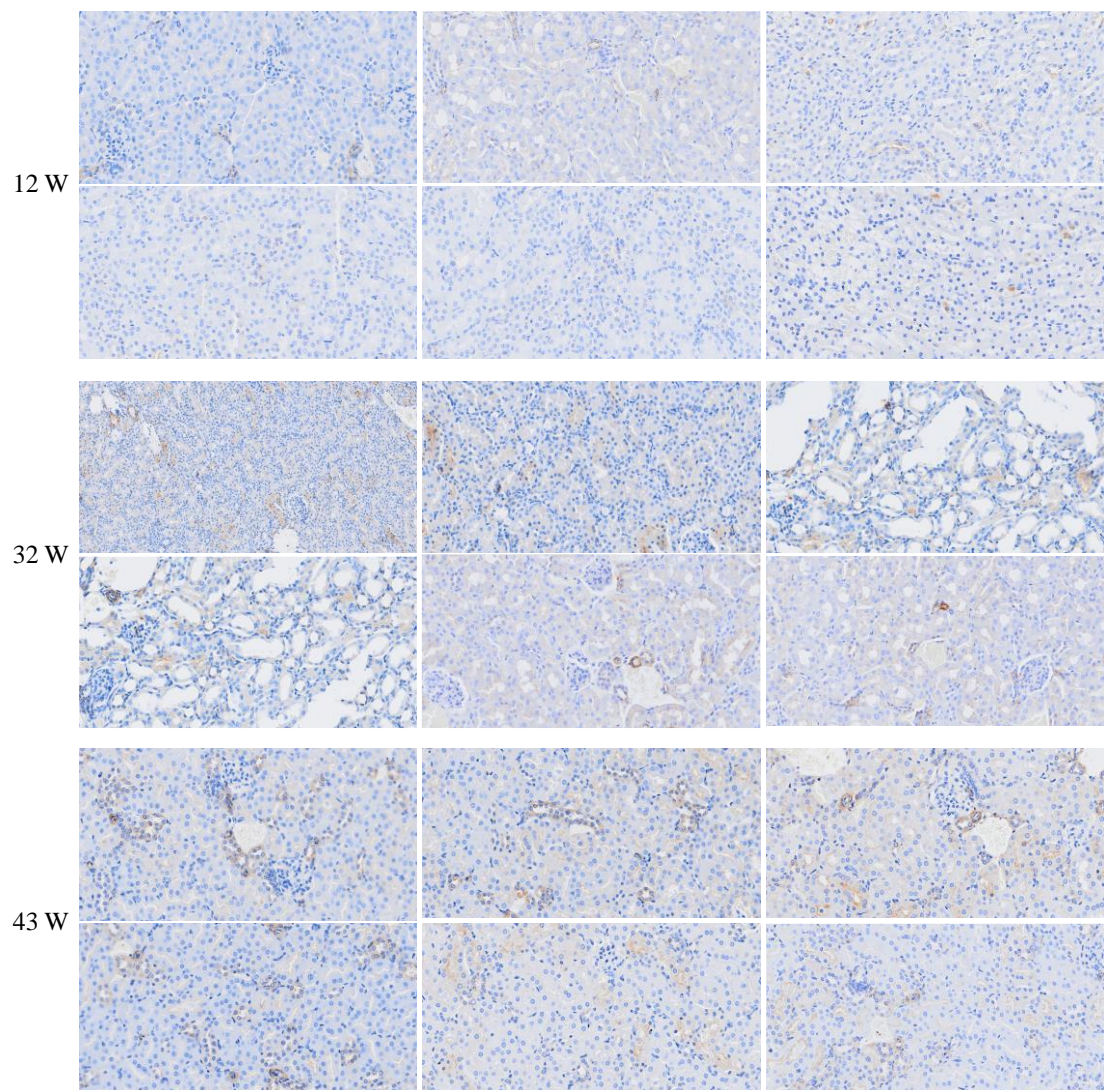

**S2 Fig. Results of  $\alpha$ -SMA staining in all mice.**

Supplement: S2 Fig — (PDF) [file pone.0315437.s004.pdf]

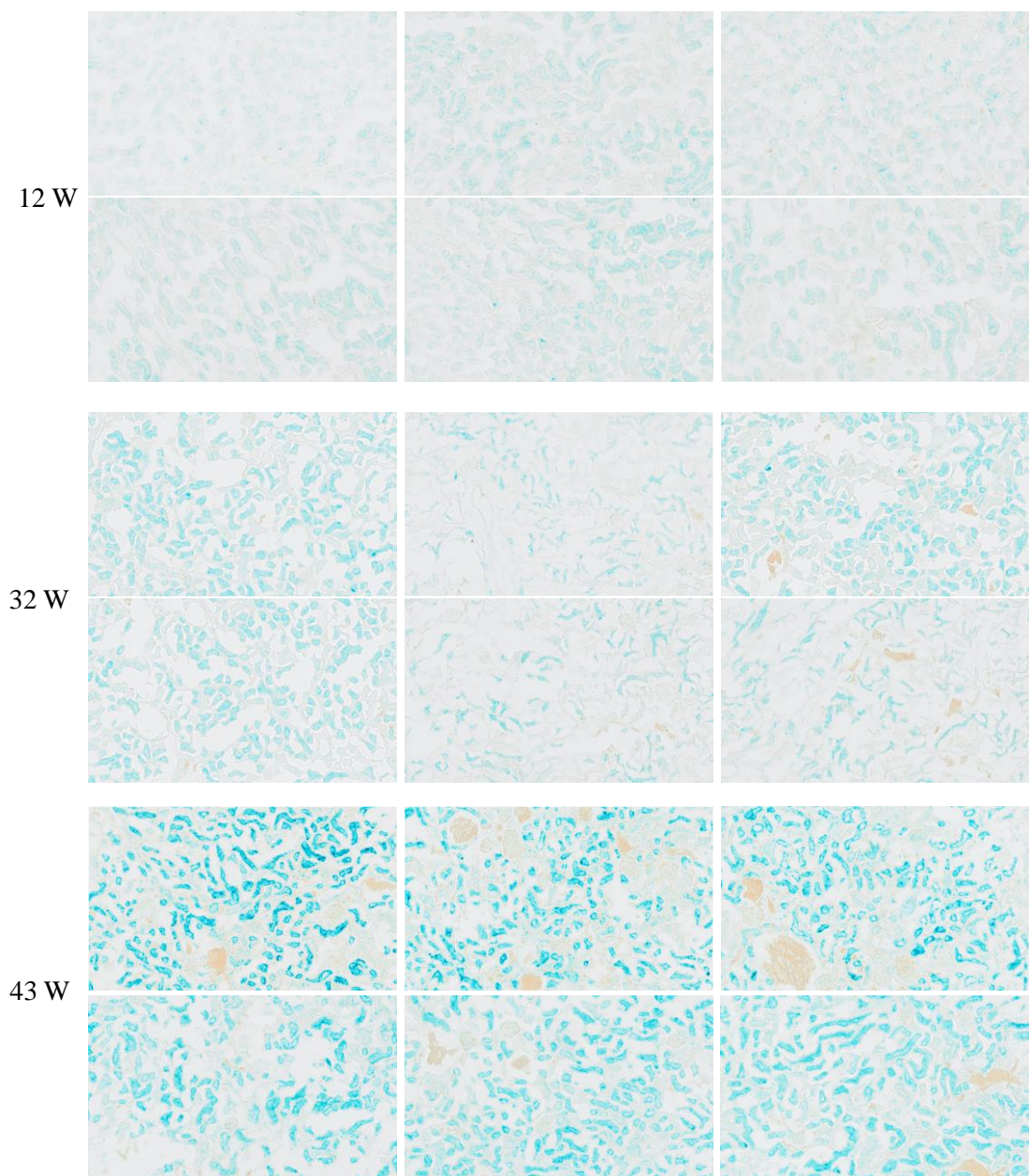

**S3 Fig. Results of SA-β-gal staining in all mice.**

Supplement: S3 Fig — (PDF) [file pone.0315437.s005.pdf]
